# Lake Malawi cichlid evolution along a benthic/limnetic axis

**DOI:** 10.1002/ece3.633

**Published:** 2013-06-07

**Authors:** C D Hulsey, R J Roberts, Y-H E Loh, M F Rupp, J T Streelman

**Affiliations:** 1 Department of Ecology and Evolutionary Biology, University of TennesseeKnoxville, Tennessee, 37996; 2 School of Biology, Institute of Bioengineering and Bioscience, Georgia Institute of TechnologyAtlanta, Georgia, 30332

**Keywords:** Adaptive radiation, African Great Lakes, aquatic locomotion, functional morphology, mbuna, utaka

## Abstract

Divergence along a benthic to limnetic habitat axis is ubiquitous in aquatic systems. However, this type of habitat divergence has largely been examined in low diversity, high latitude lake systems. In this study, we examined the importance of benthic and limnetic divergence within the incredibly species-rich radiation of Lake Malawi cichlid fishes. Using novel phylogenetic reconstructions, we provided a series of hypotheses regarding the evolutionary relationships among 24 benthic and limnetic species that suggests divergence along this axis has occurred multiple times within Lake Malawi cichlids. Because pectoral fin morphology is often associated with divergence along this habitat axis in other fish groups, we investigated divergence in pectoral fin muscles in these benthic and limnetic cichlid species. We showed that the eight pectoral fin muscles and fin area generally tended to evolve in a tightly correlated manner in the Lake Malawi cichlids. Additionally, we found that larger pectoral fin muscles are strongly associated with the independent evolution of the benthic feeding habit across this group of fish. Evolutionary specialization along a benthic/limnetic axis has occurred multiple times within this tropical lake radiation and has produced repeated convergent matching between exploitation of water column habitats and locomotory morphology.

## Introduction

**Figure 1:**
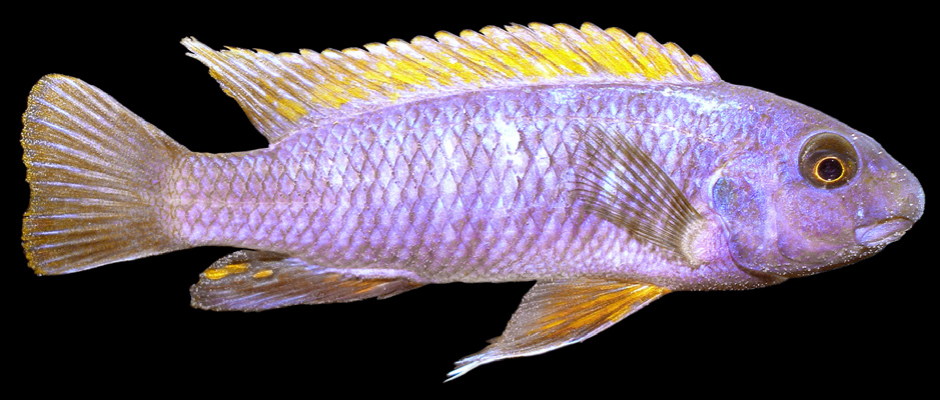
*Labeotropheus trewavasae* is one of the most recognizable of the several hundred endemic cichlids that have diversified within Lake Malawi.

Aquatic organisms frequently diversify along a benthic (bottom) to limnetic (midwater) habitat axis. For instance, across a wide diversity of fish, there is a predictable pattern of evolution into benthic and limnetic feeding niches coupled with changes in functional morphology (Robinson and Wilson 1994; Schluter 1996). However, most examples of this pattern have been documented at high latitudes in low diversity systems (Schluter and McPhail 1992; Schluter 1993; Svanbäck and Eklöv 2004). Divergence between benthic and limnetic forms might play a negligible macroevolutionary role in the diversification of species-rich freshwater fish clades. However, if this habitat axis is generally important to fish diversification, we would expect groups such as the African Great Lake cichlids (Fig. 1) to have diverged along this benthic/limnetic axis multiple times even within the same lake radiation and to exhibit phenotypic specialization associated with this divergence. Using a combination of phylogenetics, anatomy, and comparative methods, we test whether the Lake Malawi cichlid flock (LMCF) has undergone repeated shifts along a benthic/limnetic axis that has produced convergent changes in their pectoral fins.

Adaptively radiating groups like Hawaiian silver swords, tropical butterflies, and Anolis lizards have all diversified extensively along a habitat niche axis (Schoener 1968; Schluter 2000; Harmon et al. 2005; Devries et al. 2012). Within animal radiations, this habitat divergence has often resulted in convergent modifications to locomotory systems (Fulton et al. 2001; Higham 2007a). In the African Great Lakes, utilization of either rocky reefs or sandy regions has dominated thoughts about habitat specialization (Danley and Kocher 2001; Streelman and Danley 2003; Sylvester et al. 2010). This focus has meant that other types of habitat-mediated divergence such as benthic/limnetic splits have received less attention (but see Cooper et al. 2010). The relevance of this benthic/limnetic axis to cichlid diversification would be evident if utilization of these alternative habitats had evolved multiple times within the LMCF.

Enumerating the evolutionary transitions between benthic and limnetic habitats requires a phylogenetic hypothesis for LMCF species. However, phylogenetic relationships among benthic and limnetic members of the LMCF are generally ambiguous. Previous attempts at phylogeny reconstruction in the LMCF have proved frustrating because of the short time scale (∼2 million years) over which the entire group has diversified (Kocher et al. 1995; Genner et al. 2007; Hulsey et al. 2010). Additionally, molecular phylogenetic studies have had a difficult time resolving relationships among the LMCF due to a lack of variation in the molecular markers employed (Moran and Kornfield 1993; Kocher et al. 1995; Hulsey et al. 2010; Joyce et al. 2011). However, recent advances in the ability to obtain single nucleotide polymorphisms (SNPs) from across the genome could provide an extensive number of characters for reconstructing evolutionary relationships of the LMCF (Shaffer and Thomson 2007; Loh et al. 2008; Mims et al. 2010). These SNP markers combined with steadily accumulating genetic sequences could provide insight into the macro-evolutionary framework of benthic/limnetic habitat divergence among LMCF lineages.

If particular phenotypes were consistently associated with benthic and limnetic habitat specializations, the importance of this habitat divergence to LMCF diversification would be even clearer. Although East African cichlids have become textbook examples of convergence, this independent origin of similar phenotypes has mostly been documented in the trophic phenotypes of phylogenetically distinct clades of cichlids that are endemic to different lakes (Kocher et al. 1995; Ruber et al. 1999; Kocher 2004). Convergence within single lake radiations has only been detailed in a few cases (Ruber et al. 1999; Ruber and Adams 2001; Muschick et al. 2012). However, the size and structure of the pectoral fins often exhibits differences among closely related fish taxa that have diverged along a habitat axis. For instance, benthic morphotypes in many fish groups exhibit larger pectoral fins than pelagic species that share the same aquatic habitats (Malmquist 1992; Dynes et al. 1999). Yet, the fin musculature associated with benthic/limnetic divergence has rarely been examined, and it is unclear if the type of divergence found in simple systems would characterize a diverse clade like the LMCF. Nevertheless, if diversification has occurred repeatedly along a benthic/limnetic axis in Lake Malawi, the pectoral fins of the LMCF could exhibit within-lake convergence associated with habitat specialization.

Additionally, the capacity of phenotypes such as the pectoral fin structure and muscles to evolve independently of one another has been suggested to be key to the adaptive divergence of species rich groups like cichlid fishes (Liem 1973; Wainwright et al. 2004). In cichlids and other groups, several skeletal elements in the jaws and appendages that are integrated into the same functional systems have been shown to exhibit varying degrees of modular evolution (Albertson et al. 2005; Hulsey et al. 2006; Claverie et al. 2011; Parnell et al. 2012). Suites of muscles in the jaws and appendages might likewise exhibit the genetic and developmental underpinnings that are necessary for independent divergence during evolution (Kardon 1998; Hulsey et al. 2007; Widmer et al. 2007). In the LMCF, we might even expect all of the pectoral fin muscles to evolve independently and this divergence could be associated with locomotory differences that allow cichlids to exploit different habitats. For instance, in species modified to exploit rocky outcrops, one of the eight pectoral fin muscles could become much larger independently of the other muscles allowing unique morphological specializations not seen in other species. Alternatively, the pectoral fin abductors and adductors likely should functionally balance the forces each produces as they continually pull the fins back and forth (Thorsen and Westneat 2005). Coupled evolution of fin muscle masses might therefore reflect this functional complementarity (Hulsey et al. 2007). Also, a tight evolutionary correlation among all of the muscles could allow these structures to evolve in a concerted fashion and facilitate rapid evolutionary convergence (Wainwright et al. 2004; Hulsey et al. 2007). Distinct matching of pectoral fin specializations to particular aquatic habitat types might be especially likely if the structural components of the pectoral fins were tightly integrated.

Using a combination of approaches, we examined the evolution of pectoral fin structure and habitat divergence within Lake Malawi cichlids. First, we reconstructed the phylogeny of 24 LMCF habitat specialists, to ask whether a transition between benthic and limnetic habitat specialization has occurred multiple times within the LMCF. Second, we examined whether pectoral fin muscle masses and fin area have evolved independently or have generally evolved as a largely integrated unit within the LMCF. Finally, we determined whether there is an association between pectoral fin musculature and habitat divergence to test whether modifications in this important set of locomotory phenotypes have repeatedly been associated with diversification of the LMCF along the benthic/limnetic habitat axis.

## Methods

### Phylogeny

In this study, we examined the relationships and morphology of 24 species from the LMCF. Each species was designated as benthic or limnetic based on published observations of the species feeding in Lake Malawi (Reinthal 1990; Konings 2007). For the genetic analyses, all tissues utilized were reported in previous molecular studies of Malawi cichlids (Hulsey et al. 2007; Loh et al. 2008). Collection localities for the phylogenetic analyses are available from the corresponding author. To generate an improved phylogenetic hypothesis for the relationships among the 24 Malawi species examined, we examined gene sequence data from five genetic partitions as well as data from 65 SNP loci. Sequences of the mitochondrial nd2 and control region as well as the s7 intron 1, mitfb, and dlx2 nuclear partitions for all species examined were sequenced using published primers (Kocher et al. 1995; Chow and Hazama 1998; Won et al. 2005). The Genbank numbers for the new control region sequences are (KC999062-KC999079), and Genbank numbers for the remaining sequences are available in Hulsey et al. (2010, 2013). The 65 SNPs were among those ascertained from the shotgun sequencing of the genomes of five Lake Malawi cichlids and then genotyped in the cichlid species using a Beckman Coulter SNPstream™ technology (Beckman Coulter, Inc., Fullerton, CA) as described in Loh et al. (2008). The single individual used to generate sequence reads for the five loci for each species was the same individual used for SNP genotyping.

The genetic data was initially analyzed to generate three distinct phylogenetic hypotheses to determine the influence of the different data types on our phylogenetic inferences. We therefore performed similar but separate analyses of the two mitochondrial regions, three nuclear loci, and the SNPs. For the mitochondrial and nuclear sequence based phylogenies, the sequences were initially aligned using Clustal X (Larkin et al. 2007). Their alignments were then concatenated using Mesquite (Maddison and Maddison 2010). For the phylogenetic analyses, jModelTest (Posada 2008) was used to identify the best model of molecular evolution for each sequence partition. The nd2 locus was partitioned into individual codon sites, but a single partition was used for the other loci. Bayesian analyses were then executed to find approximations of the maximum likelihood tree using MrBayes 3.2 (Ronquist et al. 2012). The analyses treated the transition–transversion matrices, number of invariant sites, and gamma shape parameters as unlinked or independent for each partition. Flat prior probability distribution for all parameters were assumed before analysis. We ran six separate Bayesian analyses for 5000,000 generations with four Markov chains in each run. We sampled trees from the Markov Chain Monte Carlo (MCMC) search algorithm every 1000 generations. At the end of each analysis, the log–likelihood scores were plotted against generation time to identify the point at which log likelihood values reached a stable equilibrium. In all sets of six runs, the equilibrium appeared to be reached at approximately 100,000 generations, and therefore, sample points prior to generation 200,000 in each run were discarded as “burn–in” samples.

We also examined our SNPs in a phylogenetic context using a novel coding scheme for this data type. For each SNP allele, data was coded as 0 (homozygous), 1 (heterozygous), or 2 (homozygous for the other base) based on a random assignment of one homozygous nucleotide being coded as 0. For instance, AA would be coded as 0, AT would be coded as 1, and TT coded as 2. Our rationale for coding heterozygous sites as a distinct state is that being heterozygous at a SNP locus could be thought of as a character condition for a species. If the one individual sequenced is heterozygous, that species can be characterized as having both SNP alleles. Individuals in the population would likely be homozygous at that locus, but the species as a whole would be clearly polymorphic. Importantly, this type of retention of ancestral polymorphism is likely rampant in many groups of organisms (Moran and Kornfield 1993; Maddison 1997; Maddison and Knowles 2006). Furthermore, only 15.1% of SNPs scored were polymorphic. Additionally, our SNP character coding allowed us to analyze this data in conjunction with our more traditional analyses of sequence data. We analyzed all of the SNP coded characters as a single dataset in MrBayes 3.2 (Ronquist et al. 2012). A key feature of MrBayes is its incorporation of the Lewis (2001) MK model. This model was described for binary morphological data, where it is important to take into account the bias associated with only sampling variable sites. Our SNP data are variable in this way.

The five sequence regions were also combined with the 65 SNPs to generate a single concatenated phylogenetic hypothesis. For the three separate analyses as well as the concatenated analysis, percentages of trees that recovered a particular clade, the clade's posterior probability (pp), were depicted on the single best likelihood tree topology recovered. We also examined the effective sample size (ESS) of the likelihoods of each phylogenetic analysis remaining postburn using Tracer v1.5 (Drummond and Rambaut 2007) to ensure that values were over 200 thereby ensuring the phylogenetic searches were well mixed.

### Morphometrics

**Figure 2:**
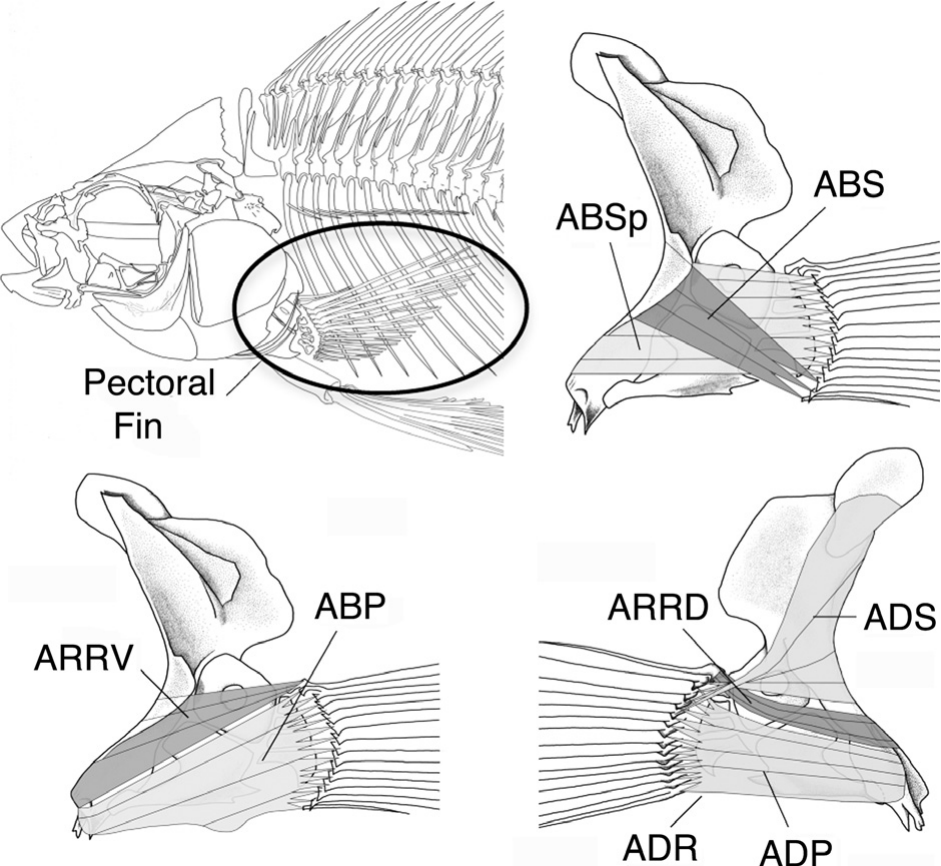
The skeletal elements of a generalized cichlid's pectoral fin and its eight pectoral muscles are depicted. We examined the mass of the major abductor muscles that included the abductor superficialis (ABS) and abductor superficialis pars profunda (ABSp). The more medial arrector ventralis (ARRV) and abductor profundus (ABP) were also dissected. These four abductor muscles all function to pull the pectoral fin anteriorly. We also examined the mass of four adductors that included the arrector dorsalis (ARRD), adductor radialis (ADR), adductor medialis (ADM), and adductor superficialis (ADS) that all pull the cichlid pectoral fin posteriorly.

The standard length (SL) that extends from the anterior tip of the jaw to the base of the caudal fin was measured for all specimens prior to dissections. For the pectoral muscle measurements (Fig. 2), the right pectoral girdle of all individuals was examined. The pectoral girdle was isolated from the body, skinned, and pectoral muscles separated from the girdle using forceps under a dissecting microscope. Each muscle was removed from its origin and cut along the tendons at its insertion onto the fin rays. Nomenclature for the pectoral musculature follows Geerlink (1983) and Thorsen and Westneat (2005).

We first determined the mass of the four large pectoral fin abductor muscles that included the abductor superficialis (ABS), abductor superficialis pars profunda (ABSp), arrector ventralis (ARRV), abductor profundus (ABP) that all pull the fin anteriorly. We also examined the mass of four pectoral adductors that included the arrector dorsalis (ARRD), adductor radialis (ADR), adductor medialis (ADM), and adductor superficialis (ADS) that together function to pull the pectoral fin posteriorly. Once removed, the muscles were stored in labeled vials of 70% ethanol. To measure their wet mass, the muscles were blotted dry on paper towels twice and their mass was subsequently quantified using a Mettler H10W balance with a precision of 0.0001 g. To describe variation in the muscle masses among individual species, the percent contribution of the mass of each individual muscle to the total pectoral muscle mass of a species was calculated. All of these percentages were also averaged to determine the general pattern of the relative masses of the eight individual muscles.

To measure pectoral fin area, the entire pectoral girdle connected to the fin was first placed for 24 h in a digestion of 5% tryspin, 30% aqueous saturated sodium borate, and 65% water. The fins were then exposed for 1 h to a 1% potassium hydroxide aqueous solution combined with 20 mg of alcian red stain. This allowed us to readily visualize the individual fin rays. The fins were then pinned into a naturally splayed position on top of waterproof paper. A digital image of the fin with a ruler in frame for calibration was obtained and imported into ImageJ (Schneider et al. 2012). Only pectoral fins that were not heavily damaged following collection in Africa, transport to the lab, and subsequent storage were utilized. Unfortunately, this resulted in the inability to use the fin areas in the full set of species and analyses. However, the correlation of fin area with total fin muscle masses suggested the muscles likely provide highly informed patterns of fin area evolution (see below). Using digital images, we outlined the external area of each pectoral fin. This outline circumscribed a region that began at the proximal end of the leading fin ray, traced along the tips of the remaining fin rays, and was completed using a line that ran across the radials from the proximal end of the final lagging fin ray to the proximal end of the leading fin ray. The region within this circular trace was measured as the pectoral fin area.

### Comparative analyses

To highlight the number of inferred transitions between water column habitats, the ancestral states of the benthic and limnetic phenotypes were mapped onto the single best phylogeny inferred from the concatenated data. Habitat evolution across the tree was inferred using likelihood reconstruction and equal transition probabilities between the two habitat types in the program Mesquite (Maddison and Maddison 2010). To perform a series of comparative analyses on the morphological data, we analyzed the influence of variation in phylogenetic reconstructions using a total of 1500 phylogenetic hypotheses. We assembled this set of trees from the 500 best phylogenetic hypotheses recovered from analyses of the mitochondrial loci, SNPs, and concatenated data. The phylogenies inferred from the nuclear sequences analyzed in isolation were not used in the comparative analyses because of the low resolution provided from these reconstructions (see below).

To examine the evolutionary associations among the muscles in a phylogenetic framework, the cube root of the individual muscle masses and total muscle mass were first determined. This linearized the masses to the same dimension as SL. Because of the influence of SL on the muscle masses, we then implemented the “phyl.resid” function in the “phytools” package (Revell 2012) in R (R Development Core Team 2012) that computes phylogenetically size-corrected values of traits for comparative analyses (Revell 2009, 2010). We generated these size-corrected values for each of our 1500 phylogenetic hypotheses. Phylogenetic independent contrast (PIC) correlations (Felsenstein 1985) between pairs of the size-corrected muscle masses were then examined on all 1500 phylogenies to determine if individual muscle masses showed extensive coevolution. A “Holm” correction for multiple comparisons was used to adjust *P-*values for the 28 pairwise correlations of the eight individual muscle masses. We also examined correlations between the absolute value of each trait's contrasts and its standard deviation using the PDAP module (Midford et al. 2008) as implemented in Mesquite (Maddison and Maddison 2010). These correlations were examined on a subset of our trees (10 trees from each of the three phylogenetic data sets) in order to ensure the contrast values were adequately standardized (Freckleton 2000; Revell 2010).

Because the sizes of the individual pectoral fin muscles were generally correlated (see below), we examined only the association between the total pectoral fin muscle masses and fin area. First, we square root transformed the fin areas to linearize them to the same dimension as SL. After testing for the correlations between the absolute value of the total pectoral fin muscles mass contrasts and standard deviation on the 30 trees used above, we ran a PIC correlation between total pectoral muscle mass and fin area across all 1500 trees. Using the results from each of the three phylogenetic data sets, we generated summary statistics for this correlation.

The evolutionary relationship between water column habitat a species uses when feeding (a categorical variable) and pectoral fin total muscle mass (continuous variable) was also examined across the LMCF phylogeny. To examine this association, we implemented a phylogenetic ANCOVA through simulation (Garland et al. 1993) in R. We wrote custom scripts that simulated evolution of total pectoral muscle mass while retaining the LMCF flock mean and variance in pectoral muscle mass as well as the evolutionary covariance structure between the total muscle mass and SL. From these analyses, we obtained simulated *F* statistics that were used to create a null distribution against which our empirical *F* statistics were tested as suggested in Garland et al. (1993). We ran these simulations 1000 times for each of the 1500 phylogenies. For each individual phylogeny, we used the proportion of the 1000 simulated *F* statistics that were greater than the empirical *F* statistic to generate *P*-values. For the three sets of phylogenies, we report the mean and standard error of the 500 *P*-values.

## Results

### Phylogeny

**Figure 3:**
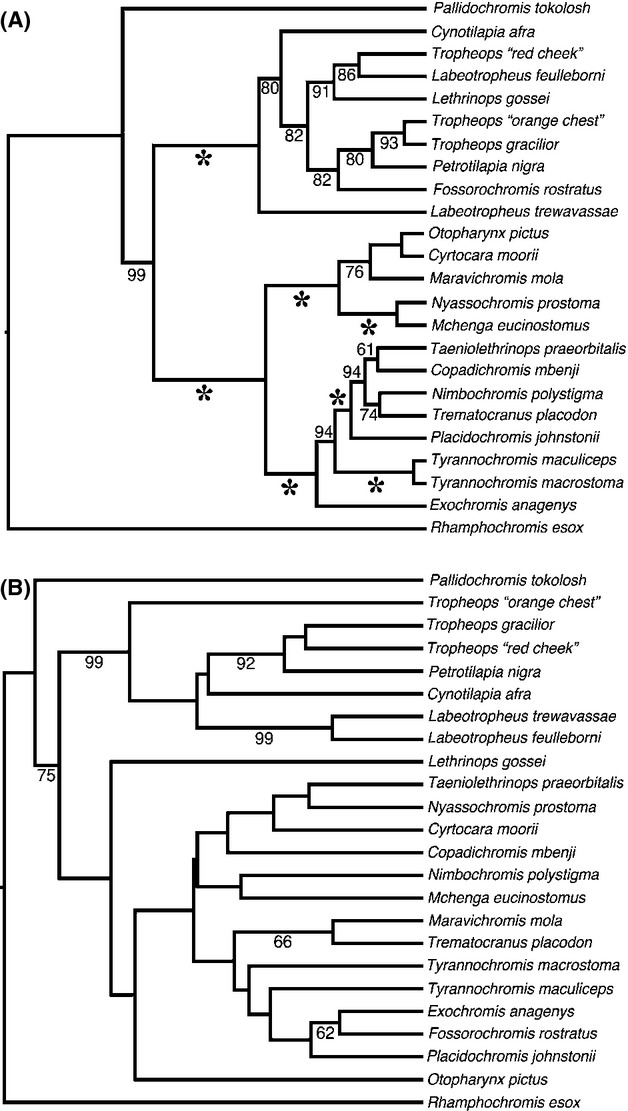
Phylogenies inferred from each of the three major data sets. Posterior probability support for individual nodes is given and nodes with 100% inferred posterior probability are depicted with an asterisk (*). The mitochondrial loci nd2 and control region generally provided the most thoroughly resolved phylogenetic hypothesis (A) for the 24 Lake Malawi cichlid species examined. The phylogeny inferred from the concatenation of the three nuclear loci s7 intron 1, mitfb, and dlx2 (not shown) did not provide much phylogenetic resolution. The phylogeny inferred from the 65 nuclear SNPs (B) provided more resolution than the nuclear sequence data.

**Figure 4:**
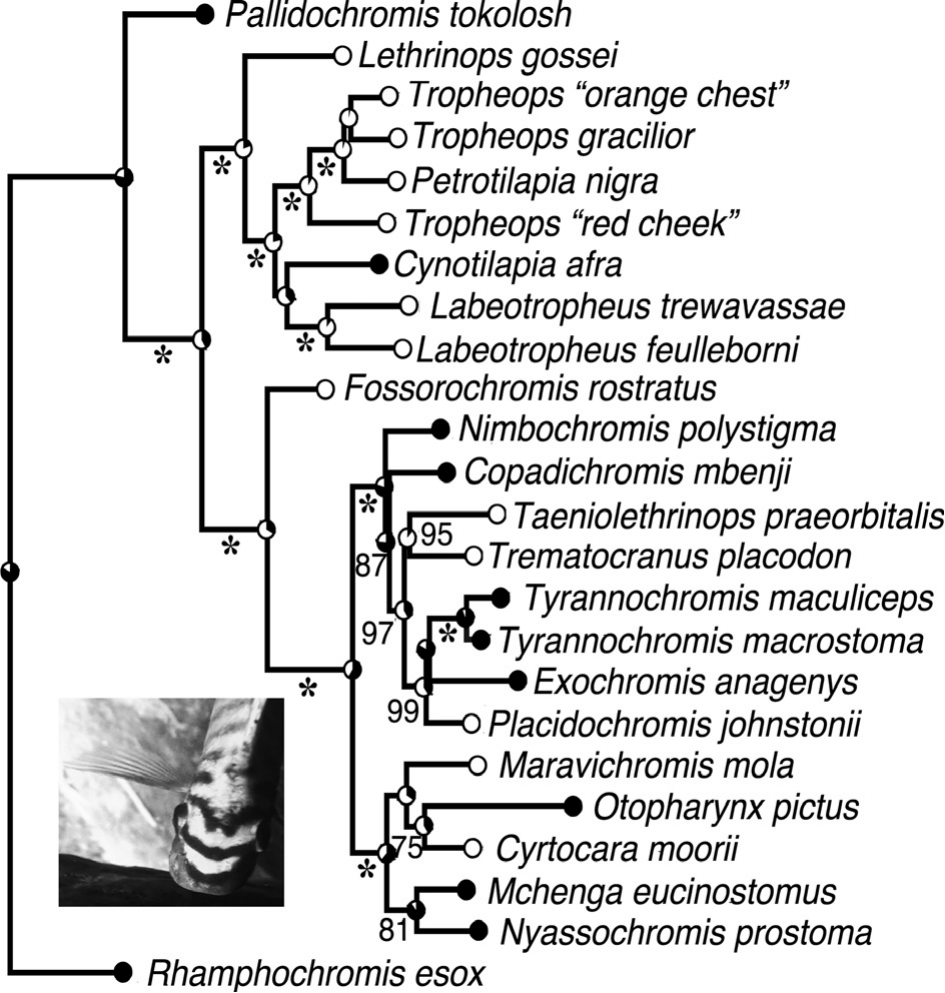
Phylogeny of Malawi cichlids reconstructed using a concatenated data set of two mitochondrial gene regions, three nuclear genes, and 65 single nucleotide polymorphisms (SNPs). The Bayesian support values for each node are shown and posterior probabilities with an asterisk (*) depict nodes with 100% posterior support. Fish were coded as benthic (white) or limnetic (black) and the ancestral states at each node in the phylogeny were reconstructed (shown as a pie diagram) to estimate the proportional likelihood of the two ancestral habitat conditions at the nodes. A dorsal view of the benthic feeding *Labeotropheus fuelleborni* and its pectoral fin in mid stroke during feeding is pictured.

The nd2 gene was 1047 base pairs (bps) in length and the control region sequences utilized were 438 bps long. Every s7 sequence was 464 bps in length. The Malawi mitfb sequences ranged from 395 to 404 bps and the dlx2 gene region ranged from 898 to 902 bps. The length of the concatenated alignment for all five sequence partitions was 3255 sites. Based on jModelTest results, first codon positions in nd2 were analyzed using the TPM3uf model of molecular evolution and nd2 third positions were analyzed with the TrN model. The dlx2 gene was modeled using TIM2. All other genetic partitions were analyzed with the Hasegawa, Kishino, and Yano model of molecular evolution. In total, there were 120 variable sites in the concatenated molecular sequence data of five genes indicating that the 65 SNPs comprised over one-third of the phylogenetically informative sites in the phylogeny. *Rhamphochromis esox* is often thought to form one of the first lineages splitting off from the rest of the Malawi cichlids (Kocher et al. 1995; Hulsey et al. 2013). Therefore, this species was used to polarize our depictions of phylogeny (Figs. 3, 4).

The mitochondrial gene trees provided the most resolution of the three individually partitioned phylogenetic analyses (Fig. 3A). There were 20 nodes that showed greater than 50% pp support. Interestingly, *Fossochromis rostratus* and *Lethrinops gossei* were found to nest with the mbuna with 100% pp based on these mitochondrial loci. There was little support for any relationships in the nuclear sequence tree (not shown) likely because of the generally low sequence divergence in the three nuclear loci (Hulsey et al. 2010). However, the three nuclear sequences did provide support for the monophyly (100% pp) of the two *Tyrannochromis* species. The SNP trees (Fig. 3B) provided better phylogenetic resolution than the ∼1850 base pairs from the three nuclear sequences. Six nodes in the SNP tree exhibited greater than 50% pp. Notably, the SNP reconstructions inferred the mbuna clades *Tropheops*, *Petrotilapia*, *Cynotilapia*, and *Labeotropheus* to be monophyletic with substantial posterior support (99%). Because of their variability, the mitochondrial loci and SNPs likely provided most of the structure for our concatenated phylogenetic analyses.

The relationships among the LMCF species inferred from the concatenated reconstructions of all of our data were generally well supported (Fig. 4). The mbuna species sequenced formed a monophyletic clade. As was generally recovered in the SNP trees, the two species of herbivorous *Labeotropheus,* were strongly supported as monophyletic (100% pp). The planktivorous *Cynotilapia afra* had ambiguous relationships within the otherwise benthic feeding mbuna. Within this mbuna clade there is also scant support for clearly defined relationships among the three members of the genus *Tropheops* and *Petrotilapia nigra* although they were supported as a monophyletic clade. The species *L. gossei* was strongly supported as more closely allied to the mbuna than to the other LMCF species sampled.

*Fossorochromis rostratus* plus a large clade containing taxa generally referred to as utaka, or sand dwellers, is strongly supported as sister to the largely mbuna clade. Within this utaka clade there is a basal split between two groups. One clade contains the planktivores *Nyassochromis protsoma*, *Mchenga eucinostomus*, and *Otopharynx pictus* as well as the benthic feeding species *Cyrtocara moorii* and *Maravichromis mola*. The other clade contains several water column feeding piscivores including *Nimbochromis polystigma*, two species of *Tyrannochromis* and *Exochromis anagenys* as well as the planktivore *Copadichromis mbenji*. Interspersed between these species were the benthic feeding *Taeniolethrinops praeorbitalis*, *Trematocranus placodon*, and *Placidochromis johnstonii*. The pelagic piscivorous predators *Pallidochromis tokolosh* and *R. esox* were supported as sister to all the remaining species examined here from the LMCF.

### Morphometrics

The four pectoral fin abductors on average (*μ*) totaled 47.8% of the mass of the eight pectoral fin muscles (Table 1). The ABP (*μ* = 16.1%, *Range*: 13.3–19.6%) and ABSp (*μ* = 14.5%, *Range*: 6.0–17.9%) generally composed the largest proportion of the pectoral fin abductor muscle mass. The ABS (*μ* = 9.2%, *Range*: 4.9–13.2%) and ARRV (*μ* = 8.0%; *Range*: 5.9–10.9%) contributed less to the total muscle mass. The ADP was consistently the largest of the four adductors and composed on average 29.1% (*Range*: 25.2–31.8%) of the total muscle mass. The ADS composed on average 17.7% (*Range*: 10.8–22.2%) of the total pectoral fin muscle mass with the ARRD (*μ* = 4.1%; *Range*: 2.2–8.3%) and ADR (*μ* = 1.3%, *Range*: 0.1–2.7%) contributing less to the total muscle masses.

**Table 1:** Habitat specialization and morphometrics of the Malawi species examined

Species	*n*	H	SL	ABS	ABSp	ARRV	ABP	ADS	ARRD	ADP	ADR	Area
*Copadichromis mbenji*	3	L	88.1	10.1	13.5	7.5	14.7	18.7	3.5	27.7	0.8	354.2
*Cynotilapia afra*	3	L	75.0	7.1	8.9	4.4	8.5	6.2	3.5	18.3	0.7	213.7
*Cyrtocara moori*	2	B	133.0	15.1	53.3	26.2	45.2	64.9	13.8	88.1	3.8	–
*Exochromis anagenys*	1	L	172.2	45.2	68.5	40.7	77.0	80.3	22.4	127.0	8.3	800.6
*Fossochromis rostratus*	3	L	76.4	5.6	8.3	6.1	8.2	10.1	1.3	15.8	0.6	159.0
*Labeotropheus fuelleborni*	4	B	97.5	20.3	31.0	10.9	31.2	29.7	6.1	53.8	2.2	360.3
*Labeotropheus trewavasae*	3	B	86.9	7.3	13.0	4.4	10.2	14.4	2.7	20.4	2.0	192.4
*Lethrinops gossei*	3	B	115.2	31.9	52.0	38.6	65.7	67.7	9.7	97.8	4.6	–
*Maravichromis mola*	2	B	113.1	16.8	35.5	19.4	30.8	50.4	9.9	66.5	2.6	–
*Mchenga eucinostomus*	4	L	81.8	6.0	7.7	5.0	11.4	11.0	2.7	19.1	0.6	96.7
*Nimbochromis polystigma*	4	L	88.2	9.9	15.8	8.1	17.1	18.0	4.2	32.1	1.1	241.4
*Nyassochromis prostoma*	3	L	114.4	19.2	36.2	22.7	38.4	43.8	8.9	69.7	2.3	367.4
*Otopharynx pictus* ”Maleri”	3	L	82.7	5.2	10.3	4.8	9.8	13.6	2.1	21.5	1.2	258.7
*Petrotilapia nigra*	3	B	103.4	21.0	30.8	14.6	35.8	30.5	6.8	60.5	1.9	–
*Placidochromis johnstonii*	1	L	130.1	25.7	41.3	19.3	49.4	53.5	15.8	96.3	5.2	–
*Placidochromis tokolosh*	1	L	145.5	26.7	16.3	27.5	48.8	55.3	8.9	83.2	5.8	–
*Rhamphochromis esox*	1	L	119.7	4.0	7.4	3.2	7.3	10.5	1.4	13.0	0.4	–
*Taeniolethrinops praeorbitalis*	3	B	139.0	43.3	66.7	37.0	86.5	81.2	15.7	152.8	7.4	760.0
*Trematocranus placodon*	3	B	131.9	29.4	52.7	30.3	51.0	66.9	18.6	103.5	6.1	–
*Tropheops gracilior*	2	B	70.3	10.9	9.9	5.7	12.8	14.3	3.0	25.7	0.7	216.9
*Tropheops “orange chest”*	3	B	83.0	11.3	18.4	7.3	17.3	18.0	4.1	31.1	1.3	250.0
*Tropheops “red cheek”*	3	B	76.9	6.9	14.0	4.8	12.9	12.2	3.9	22.6	0.7	219.6
*Tyrannochromis macrostoma*	3	L	91.9	7.5	13.6	10.2	16.8	15.4	7.9	24.3	0.9	140.5
*Tytannochromis maculiceps*	3	L	115.7	17.0	26.6	18.9	40.1	31.9	8.1	58.6	3.2	390.8

The sample sizes (*n*) and specialization of benthic (B) and Iimnetic (L) feeding habitats is noted. The standard length (SL) and masses (mg) of the pectoral muscles examined are given. The area (mm^2^) of the pectoral fin for the species examined is also given.

### Comparative analyses

For the size-corrected pectoral fin phenotypes, the absolute value of contrasts and their standard deviation were generally uncorrelated. The average *P*-values of this correlation for our traits across the 30 trees examined ranged from an average of *P* = 0.34 to *P* = 0.10 suggesting our transformations were adequate for subsequent analyses (Freckleton 2000). Our analyses of the phylogenetically size-corrected masses of individual pectoral fin muscles found them to be generally highly correlated during the evolution of the LMCF (Table 2). However, the ARRD and ADR were frequently not found to have a high correlation with the other pectoral fin muscles. The total muscle mass of the pectoral fins was also highly correlated with fin area regardless of whether we examined the mitochondrial trees (PIC *r* = 0.58 ± 0.07; *P* = 0.031 ± 0.032) SNP phylogenies (PIC *r* = 0.63 ± 0.07; *P* = 0.016 ± 0.018) or concatenated phylogenies (PIC *r* = 0.57 ± 0.07; *P* = 0.029 ± 0.021).

The transition between benthic and limnetic feeding has likely occurred at least six times in the LMCF (Fig. 4). The ANCOVA analyses supported an evolutionary relationship between total pectoral fin muscle mass and feeding habitat when SL was used as covariate. The mitochondrial (*P* = 0.039 ± 0.009), SNP (*P* = 0.045 ± 0.010), and concatenated (*P* = 0.045 ± 0.010) phylogenetic datasets all recovered associations between benthic feeding specialization and the evolution of larger pectoral fin muscles in the LMCF.

## Discussion

Evolution along a benthic/limnetic axis has occurred several times within the LMCF. Shifts between feeding on the substrate versus feeding in the water column are also important for phenotypic divergence within the LMCF. Although we did not likely capture all of the benthic/limnetic splits within the LMCF, these habitat shifts cannot account for even a majority of species-pair splits within the approximately 500 species within Lake Malawi. Therefore, the importance of benthic/limnetic habitat shifts for diversification in tropical systems like Lake Malawi might still be considered relatively minor compared to more temperate systems. Yet, divergence along this water column axis does likely play a role in structuring cichlid phenotypic divergence in Lake Malawi.

The substantial correlation structure of the pectoral fin muscles and fin area indicates these structures are likely generally evolving as a single unit (Table 2). If one muscle is getting larger in the members of the LMCF, then the other muscles are more often getting larger. The general lack of modularity in these locomotory structures might be surprising in a highly diverse clade such as the LMCF. However, several other phenotypes such as oral jaw mechanical systems (Albertson et al. 2005) and the teeth on the oral and pharyngeal jaws (Fraser et al. 2009) show highly integrated phenotypes within Malawi. Muscle systems might also commonly be evolutionarily decoupled (Kardon 1998; Widmer et al. 2007). There was variation around the proportion that individual muscles composed of the total pectoral muscle masses, but in general, the muscles showed relatively little overlap in their individual percentage contribution to the total muscle mass of a species. The correlated evolution found here for fin muscles mirrors the correlated evolution of the three adductor mandibulae that function to close the jaws in the LMCF (Hulsey et al. 2007). Tight coevolution of individual muscles that power the same skeletal structures might be especially common in rapidly diverging groups like the LMCF.

**Table 2:** The independent contrast correlation matrix among all of the size adjusted masses of the pectoral fin muscles

Muscles	ABS	ABSp	ARRV	ABP	ADS	ARRD	ADP	ADR
ABS		0.64 ± 0.04	0.77 ± 0.03	0.88 ± 0.01	0.75 ± 0.03	0.39 ± 0.06	0.89 ± 0.01	0.64 ± 0.03
	**–**	0.46/0.77	0.70/0.86	0.83/0.95	0.69/0.84	0.30/0.77	0.88/0.93	0.47/0.89
ABSp	**		0.74 ± 0.03	0.76 ± 0.03	0.82 ± 0.02	0.60 ± 0.04	0.80 ± 0.02	0.47 ± 0.05
	0.044/***	**–**	0.65/0.85	0.60/0.86	0.71/0.90	0.55/0.86	0.67/0.88	0.27/0.74
ARRV	***	***		0.85 ± 0.02	0.86 ± 0.02	0.57 ± 0.05	0.81 ± 0.03	0.51 ± 0.06
	**/***	**/***	–	0.77/0.95	0.82/0.94	0.53/0.81	0.77/0.93	0.23/0.77
ABP	***	***	***		0.83 ± 0.02	0.44 ± 0.06	0.95 ± 0.01	0.65 ± 0.04
	***/***	0.006/***	**/***	**–**	0.73/0.94	0.24/0.83	0.92/0.81	0.49/0.88
ADS	***	***	***	***		0.58 ± 0.04	0.89 ± 0.01	0.64 ± 0.04
	**/***	**/***	***/***	**/***	**–**	0.44/0.83	0.81/0.95	0.47/0.83
ARRD	0.077 ± 0.050	0.003 ± 0.003	0.006 ± 0.006	0.464 ± 0.038	0.005 ± 0.004		0.52 ± 0.05	0.24 ± 0.08
	0.205/***	0.008/***	0.012/***	0.300/***	0.044/***	**–**	0.40/0.88	0.13/0.51
ADP	***	***	***	***	***	0.015^N^ ± 0.015		0.72 ± 0.03
	***/***	***/***	***/***	***/***	***/***	0.088/***	–	0.51/0.90
ADR	**	0.029^N^ ± 0.020	0.016^N^ ± 0.015	**	**	0.306 ± 0.171	**	
	0.256/***	0.033/**	0.016/***	0.338/***	0.037/***	0.580/***	0.020/***	**–**

All cells show values for the average and standard error of the 500 concatenated trees on top with the average values from the 500 mitochondrial trees separated (/) from the 500 SNP phylogeny values on the bottom. The PIC correlation coefficients (r) for each pair of size-adjusted muscles are shown in the cells on the top and to the right of the diagonal. The *P*-values are shown on the bottom and to the left of the matrix diagonal. All *P*-values less than 0.0001 are depicted with three asterisks (***). Otherwise, the values are provided. Values for the concatenated phylogeny that were determined to be non-significant at *P*-0.05 following the “Holm” correction for multiple comparisons are denoted (N).

Locomotory abilities are critical to diversification in a number of adaptive radiations (Harmon et al. 2005; Higham et al. 2007) and pectoral fin locomotion is thought to be very important in the adaptive radiation of a number of other fish groups (Webb 1984; Schluter 1993; Svanbäck and Eklöv 2004). Our results suggest that having larger and therefore more powerful pectoral fin muscles is likely to be generally important for benthic cichlid species. However, the mechanistic reasons for this association are not completely clear. Future studies should investigate whether benthic species rely more heavily on their pectoral fins when navigating complex environments and/or more limnetic species consistently make use of other locomotory structures such as their caudal fin or body during locomotion through the water column (Webb 1984; Fulton et al. 2001). It is also possible that larger pectoral muscles are critical for not only navigating but when feeding in these particular habitats. As fish that exploit benthic feeding habitats must vigorously force their trophic apparatus against the substrate to remove algae or other attached prey items (Higham 2007a,b), feeding from the benthos could often require much larger pectoral muscles. Because of the multiple functional demands placed on locomotory structures, delineating which functions are structuring morphology is often difficult (Webb 1984; Bellwood and Wainwright 2001; Fulton and Bellwood 2002). However, the repeated association of larger pectoral muscles with benthic habitat utilization in the LMCF should provide a replicated evolutionary experiment to tease apart the reasons for this phenotypic association with habitat.

Cichlid fishes are textbook examples of convergence (Fryer and Iles 1972; Kocher et al. 1995). Our results suggest that locomotory phenotypes such as the pectoral fins could exhibit equivalent degrees of divergence and convergence as do the jaws and teeth of cichlids. The LMCF habitat divergence coupled with the convergent pectoral muscle masses found here mirrors the phenotypic replication found in a number of other adaptively radiating groups that have diverged extensively along habitat niche axes (Schoener 1968; Schluter 2000; Harmon et al. 2005; Devries et al. 2012). Although they have received less attention than the jaws and other components of the trophic apparatus, locomotory systems could be just as critical to cichlid diversification.
